# Polygenic interactions with environmental adversity in the aetiology of major depressive disorder

**DOI:** 10.1017/S0033291715002172

**Published:** 2015-11-03

**Authors:** N. Mullins, R. A. Power, H. L. Fisher, K. B. Hanscombe, J. Euesden, R. Iniesta, D. F. Levinson, M. M. Weissman, J. B. Potash, J. Shi, R. Uher, S. Cohen-Woods, M. Rivera, L. Jones, I. Jones, N. Craddock, M. J. Owen, A. Korszun, I. W. Craig, A. E. Farmer, P. McGuffin, G. Breen, C. M. Lewis

**Affiliations:** 1 MRC Social, Genetic and Developmental Psychiatry Centre, Institute of Psychiatry, Psychology & Neuroscience, King's College London, London, UK; 2 Division of Genetics and Molecular Medicine, King's College London School of Medicine, Guy's Hospital, London, UK; 3 Department of Psychiatry and Behavioral Sciences, Stanford University, Stanford, CA, USA; 4 Department of Psychiatry, Columbia University and New York State Psychiatric Institute, New York, NY, USA; 5 Department of Psychiatry, University of Iowa, Iowa City, IA, USA; 6 Division of Cancer Epidemiology and Genetics, National Cancer Institute, Bethesda, MD, USA; 7 Department of Psychiatry, Dalhousie University, Halifax, Nova Scotia, Canada; 8 Discipline of Psychiatry, School of Medicine, University of Adelaide, Adelaide, South Australia, Australia; 9 CIBERSAM-University of Granada and Instituto de Investigación Biosanitaria ibs.GRANADA, Hospitales Universitarios de Granada/Universidad de Granada, Granada, Spain; 10 Department of Psychiatry, School of Clinical and Experimental Medicine, University of Birmingham, Birmingham, UK; 11 MRC Centre for Neuropsychiatric Genetics and Genomics, Neuroscience and Mental Health Research Institute, Cardiff University, Cardiff, UK; 12 Barts and The London Medical School, Queen Mary University of London, London, UK; 13 NIHR Biomedical Research Centre for Mental Health, South London and Maudsley NHS Foundation Trust and Institute of Psychiatry, Psychology & Neuroscience, King's College London, London, UK

**Keywords:** Depression, genetics, gene-environment interactions, polygenic risk scoring

## Abstract

**Background:**

Major depressive disorder (MDD) is a common and disabling condition with well-established heritability and environmental risk factors. Gene–environment interaction studies in MDD have typically investigated candidate genes, though the disorder is known to be highly polygenic. This study aims to test for interaction between polygenic risk and stressful life events (SLEs) or childhood trauma (CT) in the aetiology of MDD.

**Method:**

The RADIANT UK sample consists of 1605 MDD cases and 1064 controls with SLE data, and a subset of 240 cases and 272 controls with CT data. Polygenic risk scores (PRS) were constructed using results from a mega-analysis on MDD by the Psychiatric Genomics Consortium. PRS and environmental factors were tested for association with case/control status and for interaction between them.

**Results:**

PRS significantly predicted depression, explaining 1.1% of variance in phenotype (*p* = 1.9 × 10^−6^). SLEs and CT were also associated with MDD status (*p* = 2.19 × 10^−4^ and *p* = 5.12 × 10^−20^, respectively). No interactions were found between PRS and SLEs. Significant PRSxCT interactions were found (*p* = 0.002), but showed an inverse association with MDD status, as cases who experienced more severe CT tended to have a lower PRS than other cases or controls. This relationship between PRS and CT was not observed in independent replication samples.

**Conclusions:**

CT is a strong risk factor for MDD but may have greater effect in individuals with lower genetic liability for the disorder. Including environmental risk along with genetics is important in studying the aetiology of MDD and PRS provide a useful approach to investigating gene–environment interactions in complex traits.

## Introduction

Major depressive disorder (MDD) is a global public health problem and the second leading cause of disability worldwide (Vos *et al.* 2012). The disorder has a well-established genetic contribution, with a heritability of 37% (Sullivan *et al.* 2000). Genome-wide association studies (GWAS) on depression have typically failed to identify the specific genetic variants involved (Ripke *et al.* 2013), although two loci have recently been implicated in the CONVERGE study of Chinese women with severe MDD (CONVERGE Consortium, 2015). Many environmental risk factors also increase the risk of depression, including unemployment, social isolation and relationship stressors (Brown & Harris, 1978). Stressful life events (SLEs) can trigger new depressive episodes and childhood trauma (CT) has been shown to double the risk for depression in adulthood (Kessler, 1997; Kendler *et al.* 1999; Nanni *et al.* 2012).

Gene–environment interactions (GxEs) whereby a person inherits sensitivity to environmental factors could also play an important role in MDD (Uher, 2014). GxEs have commonly been investigated using single loci in candidate genes, for example the serotonin transporter promoter polymorphism (*5-HTTLPR*) and its interaction with SLEs in major depression (Caspi *et al.* 2003). Despite this being the most widely investigated GxE in psychiatry, numerous studies including meta-analyses have produced discrepant results (Risch *et al.* 2009; Karg *et al.* 2011; Uher, 2014). There is more consistent evidence for an interaction between *5-HTTLPR* and CT, conferring risk for persistent depression in adulthood (Karg *et al.* 2011; Uher *et al.* 2011; Brown *et al.* 2013; Fisher *et al.* 2013). Nevertheless, the conflicting results from GxE studies in psychiatry have made these findings controversial (Duncan & Keller, 2011).

Further analyses of the genetic effects on MDD have indicated that the disorder is likely to be highly polygenic, arising from the combined effect of many risk variants, each with small effect sizes (Wray *et al.* 2012; Ripke *et al.* 2013). Polygenic risk scoring can be used to test the predictive power of multiple genetic variants simultaneously. Subsets of single nucleotide polymorphisms (SNPs) from a discovery GWAS are selected according to their *p* value and weighted by their effect size to create a polygenic risk score (PRS) for each individual in an independent validation sample. The PRS can then be tested for its ability to differentiate between case and control status in the validation dataset (Purcell *et al.* 2009; Dudbridge, 2013). PRS derived using results from the largest GWAS on MDD by the Psychiatric Genomics Consortium have shown significant predictive ability for depression, explaining about 0.9% of variance in case–control samples (Ripke *et al.* 2013; Peyrot *et al.* 2014).

These findings have led to the hypothesis that GxEs in a highly polygenic trait such as MDD may involve multiple genetic variants rather than one specific locus. Indeed, interactions between polygenic scores for MDD and CT were found to increase risk for depression in the Netherlands Study of Depression and Anxiety (NESDA), accounting for 0.6% of variance in MDD status (Peyrot *et al.* 2014). The dearth of significant results in GWAS of MDD may be partially due to environmental influences not being accounted for and investigation of GxEs could provide important insights into the complex aetiology of the disorder. Here we test for interactions between polygenic risk for major depression and adult SLEs or CT in the RADIANT UK study of recurrent MDD.

## Method

### Clinical sample collection

Depression cases (*n* = 1605) were drawn from three studies previously described in the published literature. The RADIANT UK recurrent MDD sample is comprised of the Depression Case Control (DeCC) study and probands from the Depression Network (DeNT) study of affected sibling pairs (Farmer *et al.* 2004; Cohen-Woods *et al.* 2009). UK-ascertained cases from the Genome Based Therapeutic Drugs for Depression (GENDEP) study were also included. GENDEP is a prospective pharmacogenetic study of patients with unipolar depression of at least moderate severity, on a 12-week antidepressant treatment (Uher *et al.* 2010). Briefly, patients were diagnosed using the Schedules for Clinical Assessment in Neuropsychiatry Interview, according to standardized criteria (Wing *et al.* 1990). Information was recorded on patients’ worst and second worst episodes of depression in the DeCC and DeNT studies and on their current episode in the GENDEP study (Lewis *et al.* 2010). Exclusion criteria included personal or family history of other psychiatric diagnoses besides anxiety disorder (Farmer *et al.* 2004; Cohen-Woods *et al.* 2009; Uher *et al.* 2010).

Healthy controls (*n* = 1064) were available from the DeCC study and the London site of the Bipolar Affective Disorder Case–Control study (Gaysina *et al.* 2009; Lewis *et al.* 2010). Controls were screened for lifetime absence of all psychiatric disorders using the Past History Schedule (McGuffin *et al.* 1986). First-degree family history of any psychiatric disorder or a score of 10 or more on the Beck Depression Inventory at interview were further exclusion criteria (Beck *et al.* 1996; Cohen-Woods *et al.* 2009, 2010).

Replication analysis was conducted using recurrent depression cases from The Genetics of Recurrent Early-Onset Depression (GenRED) 1 study (*n* = 260), the GenRED 2 study (*n* = 270) and the Depression Genes and Networks (DGN) study (*n* = 469) (Shi *et al.* 2011; Battle *et al.* 2014). Individuals in all analyses were of white European parentage and gave written informed consent to participate. Further information on clinical samples is provided in the online Supplementary material.

### Measures

Recurrent MDD was defined according to standard criteria, as having at least two episodes of moderate severity, separated by two or more months of remission (World Health Organization, 1993; American Psychiatric Association, 1994). Number of episodes was not a requirement in the GENDEP study, although the majority of cases were recurrent (Lewis *et al.* 2010).

Whole-blood samples were collected in ethylene-diamine-tetra-acetic acid (EDTA) from depressed cases. DNA samples were collected from controls by taking blood or using buccal mucosa swabs returned via postal mail. DNA was extracted and samples of sufficient quantity and quality were genotyped on the Illumina Human610-Quad BeadChip (Illumina, Inc., USA) (Freeman *et al.* 2003; Lewis *et al.* 2010).

Adult SLEs were assessed using the Brief Life Event Questionnaire, which is a shortened version of the List of Threatening Experiences Questionnaire (LTE-Q) (Brugha *et al.* 1985). Childbirth was also included, giving a total of 12 items (Farmer *et al.* 2004) (online Supplementary material). Cases in the DeCC and DeNT studies were asked to report on whether or not they experienced each SLE in the 6 months prior to their worst episode of depression, while GENDEP cases were asked to report on the 6 months preceding the clinical trial (Keers *et al.* 2011; Fisher *et al.* 2012). Controls reported on the 6 months prior to their interview. The number of reported SLEs was summed for each individual and analysed as a quantitative variable with range 0–12. Following the LTE-Q categories, SLEs were split into those considered dependent on an individual's behaviour and those which seem independent (Brugha *et al.* 1985). Dependent SLEs included unemployment, separation, financial or legal difficulties and the birth of a baby. Independent events included personal illness, illness of a family member, death of a family member and being robbed. This gave a total of seven dependent and five independent SLEs (online Supplementary material). Mood at the time of interview was assessed using the self-report Beck Depression Inventory in the DeCC and GENDEP cases (Beck *et al.* 1996).

A subset of the sample (*n* = 240 cases, *n* = 272 controls) completed the self-report Childhood Trauma Questionnaire, which measures frequency and severity of sexual, physical and emotional abuse, physical and emotional neglect during childhood, using 25 Likert-type items (Bernstein *et al.* 2003). CT was firstly analysed as a quantitative score with range 25–125. To explore results, CT was divided into categories of none, mild and moderate/severe, according to a definition described previously in this sample (Fisher *et al.* 2013). The GenRED and DGN replication studies assessed CT with the self-report Childhood Events Questionnaire (E. Nelson and D. Levinson, unpublished observations), which is based on the US National Comorbidity Survey CT screening items (Kessler *et al.* 1997) and CT questionnaires from Washington University (Nelson *et al.* 2002). These items cover severity and frequency of sexual abuse, physical abuse and trauma (within and outside the family), and emotional neglect.

### Quality control

Standard quality-control procedures were implemented to clean genetic data, leaving 471 747 SNPs (Lewis *et al.* 2010). Principal components (PCs) were calculated using EIGENSTRAT (Price *et al.* 2006). The first two PCs reduced the genomic control parameter (*γ*) to 1.02, indicating little difference between RADIANT UK cases and controls due to population stratification or other systematic genomic effects (Lewis *et al.* 2010). Missing information on age at worst episode of depression (233 cases) and age at interview (34 controls) was replaced with the mean age at worst episode or interview in males or females as appropriate. Number of SLEs was significantly associated with age (*p* = 3.64 × 10^−8^) and sex (*p* = 0.001), with younger individuals and females reporting more SLEs. Since depressed cases were younger than controls and contained a greater proportion of females, the number of SLEs was adjusted in cases to remove bias due to age and sex. Using controls as a proxy for the general population, a linear regression of SLEs on age and sex was used to estimate their association. These regression coefficients were then used to adjust the number of SLEs in depressed cases. Dependent and independent SLEs were adjusted separately in the same manner. CT score was not associated with age or sex, so no adjustment was performed.

### Statistical analysis

Polygenic scores were constructed using summary results available online from the Psychiatric Genomics Consortium (https://pgc.unc.edu/) MDD GWAS (Ripke *et al.* 2013). The RADIANT UK sample was removed to provide an independent validation dataset and a meta-analysis of the remaining eight studies was conducted (7615 cases and 7931 controls). These discovery GWAS results were pruned for linkage disequilibrium (LD) using the *p* value informed clumping method in PLINK v1.07 (http://pngu.mgh.harvard.edu/purcell/plink/), based on the LD structure from the RADIANT UK dataset (Purcell *et al.* 2007). Clumping preferentially retains SNPs with the strongest evidence of association and removes SNPs in high LD (*r*^2^ > 0.25 within a 300 kb window, filtering for significance, PLINK-command: --clump-p1 0.5 --clump-p2 0.1 --clump-*r*2 0.25 --clump-kb 300). Subsets of SNPs were selected from the results at nine increasingly liberal *p* value thresholds (*p*_T_ < 0.0001, *p*_T_ < 0.001, *p*_T_ < 0.01, *p*_T_ < 0.05, *p*_T_ < 0.1, *p*_T_ < 0.2, *p*_T_ < 0.3, *p*_T_ < 0.4, *p*_T_ < 0.5). These sets of alleles, weighted by their log odds ratios (ORs) from the discovery study, were summarized into PRS for each individual in the validation sample using PLINK (Purcell *et al.* 2009). *p*_T_ < 0.5 contained 87 737 SNPs.

PRS for MDD were tested for ability to predict case/control status in RADIANT UK using logistic regression in R (http://www.r-project.org) to calculate the Nagelkerke's pseudo-*R*^2^ measure of variance explained, excluding the variance accounted for by two PCs (Nagelkerke, 1991). SLEs and CT score were also tested for association with case/control status. Interactions between PRS and SLEs or CT were investigated using two models. A multiplicative model tests interaction as departure from multiplicativity, meaning that the combined effect of PRS and environment differs from the product of their individual effects. This was tested using a logistic regression, co-varying for the main effects of PRS, environment and two PCs. Models were also adjusted for PC × environment and PC × PRS interactions (Keller, 2014). The interaction term was tested for its ability to differentiate between case and control status in the validation sample by calculating Nagelkerke's pseudo-*R*^2^. An additive interaction model tests whether the combined effect of PRS and environment differs from the sum of their individual effects. It has been suggested that this better captures the biological mechanism of GxEs (Rothman *et al.* 2008). Interaction as departure from additivity was tested using linear regression of MDD case/control status on the interaction term, with covariates as described previously. The multiple-*R*^2^ measure of variance explained by the interaction was calculated. To investigate gene–environment correlations, whereby genotypes may influence exposure to different environments, PRS for MDD were tested for association with SLEs or CT score using a linear regression, co-varying for two PCs. Empirical *p* values were calculated using permutation procedures for all analyses. Ten independent tests were conducted, giving a Bonferroni corrected significance threshold of 0.005. In the replication phase, gene–environment correlations between PRS and CT score in depressed subjects were tested in MDD cases from the independent GenRED and DGN samples.

Power calculations for our study were performed in QUANTO version 1.2.4 (Gauderman & Morrison, 2009), using ORs reported in the literature for the effects of PRS (OR = 1.22), 2+ SLEs (OR = 1.82) and CT (OR = 2.27) on MDD (Nanni *et al.* 2012; Motrico *et al.* 2013; Peyrot *et al.* 2014). The study had >80% power to detect an interaction between PRS and SLEs with an OR of 1.28 (at *α* = 0.05). In the subset with CT data, there was >80% power to detect an interaction between PRS and CT with an OR of 1.76 (at *α* = 0.05).

### Ethical standards

All procedures contributing to this work comply with the ethical standards of the relevant national and institutional committees on human experimentation and with the Helsinki Declaration of 1975, as revised in 2008.

## Results

### Sample characteristics

Sample characteristics are shown in Table 1. Cases contained a significantly greater proportion of females than controls and their mean age at worst episode of depression was significantly younger than mean age at interview in controls. Cases had experienced significantly more SLEs and had higher CT scores than controls (Table 1). In the subset of the sample with CT data (240 cases and 272 controls), similar differences in sex and age at interview were found between cases and controls (*p* = 2.9 × 10^−4^ and *p* = 0.004, respectively).

**Table 1.** Sample characteristics

	Cases (*n* = 1605)	Controls (*n* = 1064)	*p* ^a^
Sex, *n* (%)			6.83 × 10^−10^
Male	471 (29.3)	436 (41.0)	
Female	1134 (70.7)	628 (59.0)	
Age, years^b^	36.7 (12.3)	41.5 (13.2)	4.16 × 10^−19^
Age of onset, years	23.1 (11.4)		
Number of episodes	2.48 (0.68)		
Number of SLEs^b^	1.57 (1.48)	0.68 (0.87)	1.67 × 10^−67^
Number of dependent SLEs^b^	0.88 (1.05)	0.23 (0.51)	3.05 × 10^−79^
Number of independent SLEs^b^	0.69 (0.89)	0.44 (0.66)	1.29 × 10^−11^
Childhood trauma score^c^	46.31 (16.25)	32.75 (8.75)	7.13 × 10^−26^

Data are given as mean (standard deviation) unless otherwise indicated.

SLEs, Stressful life events.

a *p* Values were calculated using a non-parametric Mann–Whitney *U* test, with the exception of sex where a χ^2^ test was used.

b For cases at worst episode of depression and for controls at interview.

c Data were available on a subset of 240 cases and 272 controls. Statistics were calculated from individuals without missing data.

### Interaction with SLEs

**Fig. 1.**
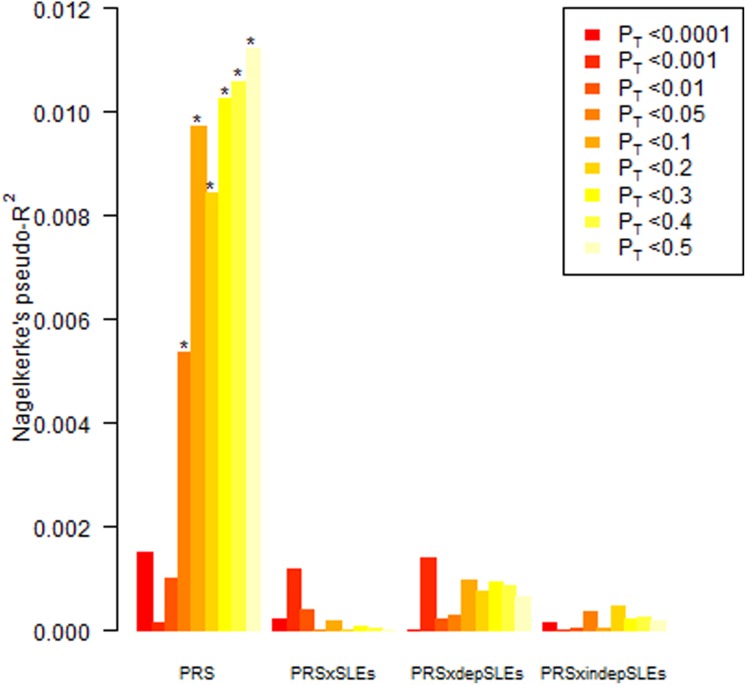
Polygenic risk scores (PRS) for major depressive disorder and multiplicative interactions with stressful life events (SLEs) used to predict depression in the RADIANT UK sample. The y-axis indicates Nagelkerke's pseudo-*R*^2^, a measure of the variance explained. On the x-axis the nine *p* value thresholds used to select single nucleotide polymorphisms in the discovery phase are plotted left to right. depSLEs, Dependent SLEs; indepSLEs, independent SLEs; *p*_T_, *p* value threshold. * *p* < 0.005. For a colour figure, see the online version.

Polygenic scores derived from a meta-analysis of MDD using data from the Psychiatric Genomics Consortium showed significant predictive ability for depression in the RADIANT UK sample. As more SNPs were added to the PRS at increasingly liberal *p* value thresholds, the amount of variance explained increased. At *p*_T_ < 0.5, the polygenic score explained 1.1% of variance in case/control status [*p* = 1.9 × 10^−6^, OR = 1.22, 95% confidence interval (CI) 1.12–1.32] (Fig. 1). After adjustment for age and sex, total SLEs were still significantly associated with MDD status (*p* = 2.19 × 10^−4^), explaining 0.7% of variance between cases and controls. A greater number of dependent SLEs was associated with case status and could predict 6.6% of variance in phenotype (*p* = 1.35 × 10^−25^). Independent SLEs showed significant but weaker predictive ability (*p* = 1.36 × 10^−9^, Nagelkerke's pseudo-*R*^2^ = 0.019) and in contrast to dependent SLEs, more independent events were found in controls *v*. cases, after correction for age and sex. Under a multiplicative model, there was no interaction between the PRS and total number of SLEs (Fig. 1). The largest *R*^2^ was 0.001 at *p*_T_ < 0.001 (*p* = 0.12, OR = 1.05, 95% CI 0.98–1.12). Interactions between PRS and dependent or independent SLEs were also non-significant (Fig. 1). No interactions were found under additive models (online Supplementary material).

### Interaction with CT

**Fig. 2.**
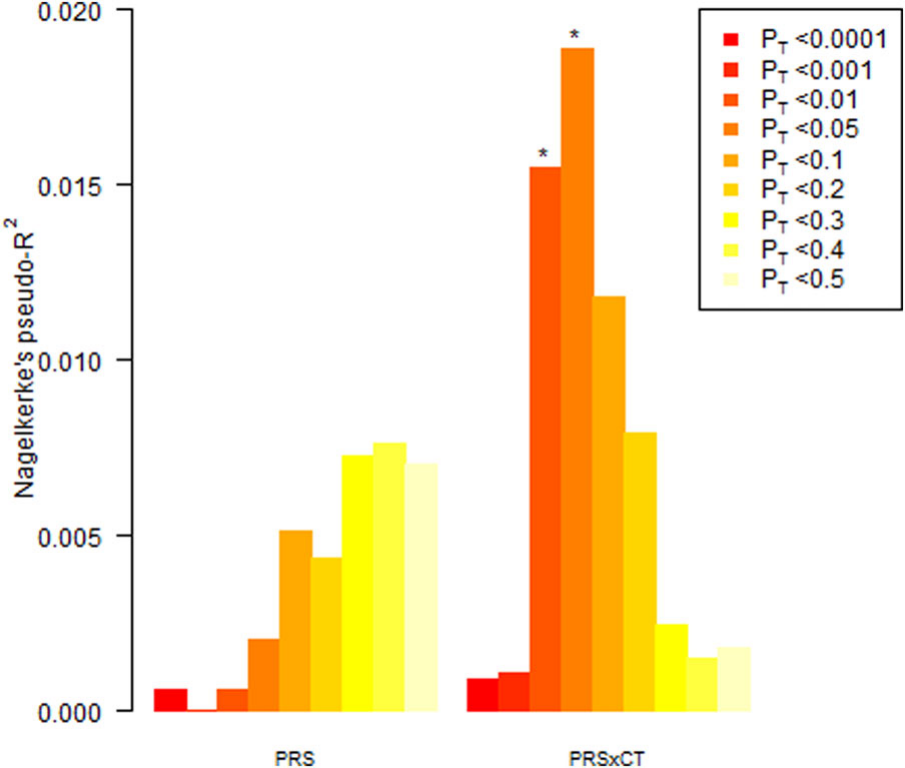
Polygenic risk scores (PRS) for major depressive disorder and multiplicative interaction with childhood trauma (CT) used to predict depression in the RADIANT UK sample. The y-axis indicates Nagelkerke's pseudo-*R*^2^, a measure of the variance explained. On the x-axis the nine *p* value thresholds used to select single nucleotide polymorphisms in the discovery phase are plotted left to right. *p*_T_, *p* value threshold. * *p* < 0.005. For a colour figure, see the online version.

**Fig. 3.**
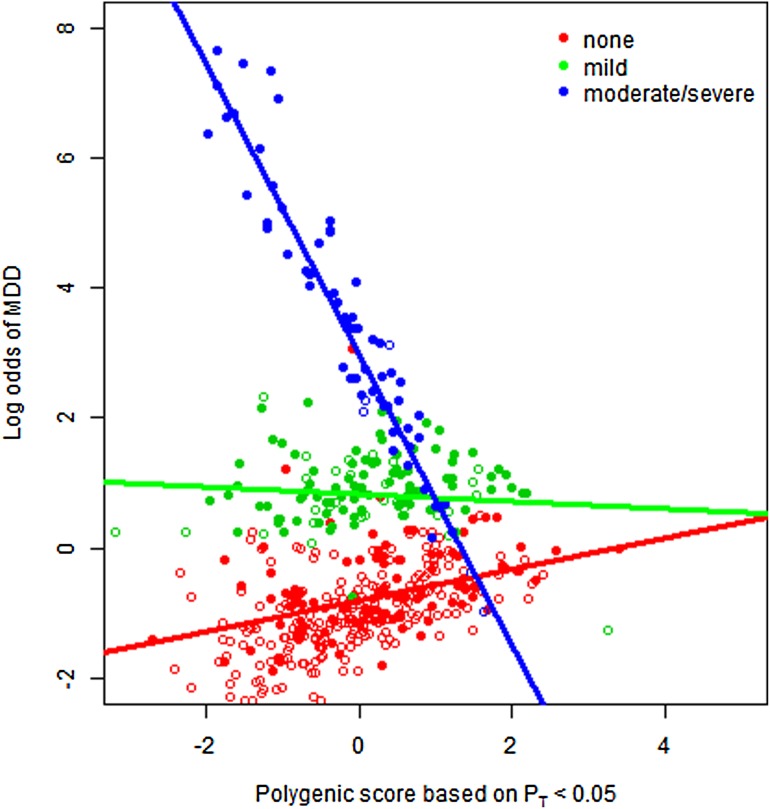
Multiplicative interaction between standardized polygenic risk score for major depressive disorder (MDD) based on *p*_T_ < 0.05 and categories of childhood trauma. Shaded circles are cases and open circles are controls. *p*_T_, *p* value threshold. For a colour figure, see the online version.

In the subset of the sample with CT data, the PRS did not show significant predictive ability for MDD (*p* = 0.078, Nagelkerke's pseudo-*R*^2^ = 0.007, *p*_T_ < 0.4), though effects were in the expected direction (OR = 1.18, 95% CI 0.98–1.42) (Fig. 2). A higher CT score was significantly associated with depression status, explaining 30.2% of variance (*p* = 5.12 × 10^−20^). Multiplicative interactions were found between polygenic scores for MDD and CT (Fig. 2). The interaction at *p*_T_ < 0.05 explained 1.9% of variance in the phenotype and was significant after multiple testing correction (*p* = 0.002). There was an inverse association between the interaction and MDD status (OR = 0.96, 95% CI 0.94–0.98). To visualize these results, interactions were plotted between categories of CT (none, mild, moderate/severe) and PRS standardized to mean 0 and s.d. 1. Plotting log odds of depression by polygenic score for each CT category at the *p* value threshold with most significant interaction (*p*_T_ < 0.05; *p* = 0.002) allows visualization of the results (Fig. 3). For individuals who had not experienced CT, a higher PRS for MDD was associated with a higher risk of the disorder (Fig. 3; black line). Individuals in the mild CT category were at an increased risk of depression but this appeared to act independently of their genetic liability (mid-grey line). Those who had experienced moderate/severe CT were mostly depressed cases but interestingly the individuals at highest risk in this category had a lower PRS than average (Fig. 3; light grey line). There were no interactions between PRS and CT under additive models (online Supplementary material).

### Gene–environment correlations

Gene–environment correlations were explored between PRS for MDD and number of SLEs. Significant correlations were found within the MDD cases, specifically with dependent (*p*_T_ < 0.001; *p* = 0.002) and not independent SLEs (online Supplementary material). No significant corrections were found between polygenic score and CT score in the RADIANT UK sample (online Supplementary material). As the interaction between PRS and CT showed an inverse association with MDD status, the relationship between PRS and CT score was tested in the independent GenRED and DGN depression cases. No significant correlations between PRS and CT score were found.

### Mood at interview

To investigate whether low mood at the time of interview may result in a recall bias for negative events, cases severely depressed at interview were removed in a sensitivity analysis. Gene–environment correlations between PRS and dependent SLEs in cases were no longer significant, excluding those who were severely depressed at interview (online Supplementary material). Interactions between PRS and CT score remained significant after severely depressed cases were excluded (online Supplementary material).

## Discussion

Polygenic scores derived from the Psychiatric Genomics Consortium MDD mega-analysis predicted depression in the RADIANT UK sample, explaining 1.1% of variance in case/control status. This modest figure is in line with previous estimates from this mega-analysis and confirms the presence of associated variants that the original GWAS was underpowered to detect (Ripke *et al.* 2013; Peyrot *et al.* 2014). SLEs were also significant predictors of case/control status. We hypothesized that given the polygenicity of MDD, testing interactions between polygenic scores and environmental adversity could be a more powerful approach than using single genetic variants in a candidate gene. No interactions were found between PRS for MDD and total, dependent or independent SLEs, which is in agreement with previous findings by Musliner *et al.* (2015).

In the subset of the sample with CT data, the PRS failed to show significant predictive ability for depression which probably reflects the restricted sample size. Consistent with previous reports, CT was a strong risk factor for recurrent MDD in adulthood (Nanni *et al.* 2012). Significant interactions were found between PRS and CT; however, there was an inverse association with depression status. This appeared to be driven by individuals who had experienced moderate/severe CT, as those at highest risk in this category tended to have a lower PRS than other cases or controls (Fig. 3). One possible explanation is that CT may be more important in the development of MDD for individuals who have a low genetic risk than for individuals who have a high genetic risk. This would be consistent with the liability threshold model for MDD, where a combined effect of many genetic risk variants together with an environmental contribution causes an individual to cross the liability threshold and become affected. Alternatively, the experience of CT may be such a strong risk factor for depression that genetics has a negligible effect.

In contrast to our results, the NESDA study found a significant PRS x CT interaction, whereby higher PRS and severe CT increased the risk for MDD (Peyrot *et al.* 2014). The conflicting results of these studies may be due to differences in design – for example, NESDA is a population-based study, includes single-episode, recurrent and chronic depression, used a different instrument for assessing CT and had a larger sample size (1645 MDD cases, 340 controls). It has been reported that interaction between *5-HTTLPR* and CT specifically increases risk for chronic depression in adulthood (Brown *et al.* 2013). This suggests that GxEs may be more specific than anticipated and subtle differences between our study and NESDA could have contributed to the discrepant results. Similarly, our SLE assessment was for the 6 months prior to the worst episode of depression in recurrent depression cases. Testing SLEs preceding the initial onset of depression or in single-episode depression may identify different components of the gene–environment aetiology of MDD.

Evidence of gene–environment correlation was found, as polygenic scores for MDD increased exposure to (or reporting of) dependent SLEs in MDD cases (online Supplementary material). This suggests that depressed individuals may select themselves into environmental adversity by creating stressful life events due to their own behaviour, which is known as active gene–environment correlation. SLEs or the reporting of SLEs is heritable (Power *et al.* 2013) and twin studies have shown pleiotropy between the genetic contribution to SLEs and genetic liability to depression (Kendler & Karkowski-Shuman, 1997; Silberg *et al.* 1999).

There are several strengths of this study. SLEs were adjusted for age and sex prior to the analyses. The amount of variance explained by the SLEs decreased dramatically after adjustment (online Supplementary material), which demonstrates the importance of accounting for age and sex. It has been suggested that low mood at the time of interview may cause recall bias for negative events; however, we found no evidence that this influenced our results, consistent with two previous analyses in the RADIANT UK sample (Fisher *et al.* 2012, 2013).

A number of limitations also warrant noting. The discovery GWAS by the Psychiatric Genomics Consortium was underpowered to detect the likely effect sizes in MDD, which reduces the ability to separate modest signals from noise and achieve accuracy in estimation of the PRS (Dudbridge, 2013; Ripke *et al.* 2013). The PRS used in these analyses consists of SNPs selected from a study of their main effect on MDD, which may not be the same genetic variants that are involved in GxEs. This could explain the non-standard shape of the PRS histograms for the interactions, in contrast to the usual pattern where variance explained increases across the *p* value thresholds (Figs 1 and 2). Our study design relied on retrospective self-reports of depression and environment, which may be less accurate if the events occurred a long time ago. However, the worst episode of depression is arguably the most memorable, and retrospective self-reports of depressive episodes agree well with hospital records (McGuffin *et al.* 1986; Kendler *et al.* 1993).

The detection of GxEs has implications for future research strategies. Analysis of cohorts with heterogeneous environmental exposures may partially explain the lack of success in detecting genetic associations with MDD. Our results suggest that more power could be leveraged from GWAS by focusing only on individuals not exposed to CT as this might identify ‘more genetic’ cases of MDD. However, results of the NESDA study suggest that focusing on exposed individuals could render genetic effects larger, more homogeneous and easier to detect (Flint & Kendler, 2014; Peyrot *et al.* 2014). Polygenic interactions in MDD require further investigation in larger, similarly well-characterized samples and could provide important insights into the complex aetiology of depression.

## Supplementary material

For supplementary material accompanying this paper visit http://dx.doi.org/10.1017/S0033291715002172. click here to view supplementary material
