# Supplementary material for: Polygenic interactions with environmental adversity in the aetiology of major depressive disorder
Source: Psychol Med. 2015 Nov 3;46(4):759–70. doi: 10.1017/S0033291715002172 (PMC4754832; doi:10.1017/S0033291715002172)
Supplement: Supplementary file 1 [file S0033291715002172sup001.doc]

**Supplementary material**

**Polygenic interactions with environmental adversity in the aetiology of major depressive disorder**

Mullins N.^1*^, Power R.A.^1^, Fisher H.L.^1^, Hanscombe K.B.^2^, Euesden J.^1^, Iniesta R.^1^, Levinson D.F.^3^, Weissman M.M.^4^, Potash J.B.^5^, Shi J.^6^, Uher R.^1,7^, Cohen-Woods S.^8^, Rivera M.^1,9^, Jones L.^10^, Jones I.^11^, Craddock N.^11^, Owen M.J.^11^, Korszun A.^12^, Craig I.W.^1^, Farmer A.E.^1^, McGuffin P.^1^, Breen G.^1,10^, Lewis C.M.^1,2^

**Corresponding Author**

Niamh Mullins MSc. - MRC Social, Genetic and Developmental Psychiatry Centre, Institute of Psychiatry, Psychology & Neuroscience, King's College London, 16 De Crespigny Park, London SE5 8AF, United Kingdom. +44 20 7848 0039. [Niamh.mullins@kcl.ac.uk](mailto:Niamh.mullins@kcl.ac.uk)

**Author Affiliations**

1. MRC Social, Genetic and Developmental Psychiatry Centre, Institute of Psychiatry, Psychology & Neuroscience, King's College London, United Kingdom
2. Division of Genetics and Molecular Medicine, King's College London School of Medicine, Guy's Hospital, London, United Kingdom
3. Department of Psychiatry and Behavioral Sciences, Stanford University, Stanford, California
4. Department of Psychiatry, Columbia University and New York State Psychiatric Institute, New York, NY, USA
5. Department of Psychiatry, University of Iowa, Iowa City, Iowa, USA
6. Division of Cancer Epidemiology and Genetics, National Cancer Institute, Bethesda, MD, USA.
7. Department of Psychiatry, Dalhousie University, Halifax, Nova Scotia, Canada
8. Discipline of Psychiatry, School of Medicine, University of Adelaide, Adelaide, South Australia, Australia
9. CIBERSAM-University of Granada and Instituto de Investigación Biosanitaria ibs.GRANADA.

Hospitales Universitarios de Granada/Universidad de Granada, Granada, Spain.

1. Department of Psychiatry, School of Clinical and Experimental Medicine, University of Birmingham, Birmingham, United Kingdom
2. MRC Centre for Neuropsychiatric Genetics and Genomics, Neuroscience and Mental Health Research Institute, Cardiff University, Cardiff, United Kingdom
3. Barts and The London Medical School, Queen Mary University of London, London, United Kingdom
4. NIHR Biomedical Research Centre for Mental Health, South London and Maudsley NHS Foundation Trust and Institute of Psychiatry, Psychology & Neuroscience, King's College London, United Kingdom.

**Table of Contents**

Clinical Sample Characteristics…………………………………………………………………………………………………………………………………….2

The Brief Life Event Questionnaire……………………………………………………………………………………………………………………………..3

Adjustment of SLEs for age and sex…………………………………………………………………………………………………………………………….4

Supra-additive interactions.………………………………………………………………………………………………………………………………………..4

Gene-environment correlations………………………………………………………………………………………………………………………………….5

Mood at Interview………………………………………………………………………………………………………………………………………………………6

References………………………………………………………………………………………………………………………………………………………………….8

**Clinical Sample Characteristics**

Participants in the DeCC and DeNT studies were identified in London, Cardiff and Birmingham from psychiatric clinics, hospitals, general medical practices and media advertisements . The GENDEP clinical trial was conducted across nine European centres and UK ascertained cases were included in these analyses . Patients were diagnosed using the Schedules for Clinical Assessment in Neuropsychiatry Interview (SCAN), according to standardised criteria in the International Classification of Diseases 10^th^ edition (ICD-10) or Diagnostic and Statistical Manual 4^th^ edition (DSM-IV) . Table S1 shows the characteristics of depressed cases from the DeCC, DeNT and GENDEP studies separately. Healthy controls in the DeCC and BACCs studies were recruited through the Medical Research Council general practice research framework, newspaper advertisements or via internal emails at King’s College London .

Depressed cases from the GenRED 1, GenRED 2 and DGN studies were used in replication analyses. Cases in the GenRED studies were recruited in clinical settings and through media and internet announcements and advertisements by six research groups at Stanford University, Columbia University, Johns Hopkins, Rush Presbyterian Medical Center Chicago, University of Iowa and University of Pittsburgh . Cases were diagnosed using the Diagnostic Interview for Genetics Studies 3.0; family informant if available was interviewed with the Family Interview for Genetic Studies and psychiatric records were obtained where possible . Cases had recurrent (≥2 episodes) or chronic (≥3 years) MDD with onset before 31 years old. All subjects were of European ancestry. GenRED 1 cases were genotyped using the Affymetrix 6.0 genome-wide SNP array (Affymetrix, Santa Clara, USA) and GenRED 2 cases were genotyped using the Illumina HumanOmni1-Quad BeadChip (Illumina, Inc., San Diego, USA). The self-report Childhood Events Questionnaire was completed for 260 GenRED 1 cases and 270 GenRED 2 cases (E. Nelson and D.Levinson, unpublished).

In the DGN study, a survey research company (Knowledge Networks, Menlo Park, CA) recruited 469 recurrent MDD cases from participants in an online survey panel that is recruited on an ongoing basis using random digit dialing of nationally-representative US households . Online screening was carried out using the Composite International Diagnostic Interview depression and alcohol and substance dependence modules . Prospective cases were selected who reported two or more episodes meeting criteria for MDD but denied lifetime substance dependence. These individuals were then interviewed using the Structured Clinical Interview for the Diagnostic and Statistical Manual of Mental Disorders IV (SCID) and those not meeting the initial eligibility criteria were excluded . Participants were all of European ancestry and were genotyped on the Illumina HumanOmni1-Quad BeadChip (Illumina, Inc., San Diego, USA) . Childhood trauma was assessed in the DGN cases using the Childhood Events Questionnaire (E. Nelson and D. Levinson, unpublished).

**The Brief Life Event Questionnaire**

Stressful life events (SLEs) were assessed in RADIANT UK using the Brief Life Event Questionnaire (bLTE-Q), which is a shortened version of the List of Threatening Experiences Questionnaire (LTE-Q) . Childbirth was added to the questionnaire to give a total of 12 items (Table S2) . Following the LTE-Q categories, SLEs were split into those considered dependent on an individual’s behaviour and those which seem independent (Table S2) .

**Adjustment of SLEs for age and sex**

Total number of SLEs was significantly associated with age (P = 3.64 x 10^-8^) and sex (P = 0.001), with younger individuals and females reporting more SLEs. As cases were younger than controls and contained a greater proportion of females, SLEs were adjusted for age and sex prior to the analyses. Using controls as a proxy for the general population, a linear regression of SLEs on age and sex was used to estimate their association. These regression coefficients were used to calculate the adjusted number of SLEs in the cases. Dependent and independent SLEs in cases were adjusted separately in the same manner. Prior to adjustment, the total number of SLEs predicted 16.3% of variance in case/control status using logistic regression. Dependent SLEs predicted 18.6% of variance and independent SLEs predicted 3.1% of variance. After adjustment for age and sex, the amount of variance explained decreased to 0.7%, 6.6% and 1.9% for total, dependent and independent SLEs respectively.

**Supra-additive interactions**

An additive model tests interaction as departure from additivity meaning that the combined effect of PRS and environment differs from the *sum* of their individual effects. This was tested using a linear regression of MDD case/ control status on the interaction term, co-varying for the main effects of PRS and environment and two PCs. Models were also adjusted for PC x environment and PC x PRS interactions . No interactions were found between polygenic score and total, dependent or independent SLEs (Table S3). No significant interactions were found between PRS and CT under the additive model (Table S4).

**Gene-environment correlations**

Gene-environment correlations were tested using a linear regression of polygenic scores for MDD on total number of SLEs, with two principal components as covariates. This was tested in the whole sample and separately in cases and controls (Table S5). In cases, significant gene-environment correlations were found, specifically with the dependent and not independent SLEs (P= 0.001, P_T_ <0.001) (Table S6).

No significant gene-environment correlations were found between PRS and CT score in the total sample, in depressed cases or controls (Table S7).

**Mood at Interview**

Mood at the time of completion of the bLTE-Q was assessed using the Beck Depression Inventory (BDI) . Data were not available on the DeNT depression cases, leaving a subset of 1254 cases. 26.2% of cases had a score of 29 or more on the BDI and were classified as severely depressed at interview. Number of SLEs was investigated in these individuals, to test whether low mood at interview was associated with a recall bias for negative events. Cases from the GENDEP study were excluded from these analyses because they were asked to report on SLEs which occurred in the 6 months prior to the clinical trial, rather than reporting retrospectively on their worst episode of depression. Individuals who were severely depressed at the time of completion of the bLTE-Q (n=286) retrospectively reported a mean of 1.93 SLEs (s.d. 1.64) in the 6 months prior to their worst episode of depression, which was significantly higher than the mean number of 1.46 (s.d. 1.37) reported by other cases (n=889) (P = 5.09 x 10^-6^). Analysis of gene-environment correlations excluding cases who were severely depressed at interview (n=889 remaining), no longer showed any significant associations between polygenic scores and dependent SLEs (Table S8), although effects were in the same direction as in the total cases (Table S6). The lack of significance may be caused by a loss in power due to removal of half the cases rather than an association between low mood at interview and reporting of SLEs.

In the childhood trauma sample, individuals who were severely depressed at the time of interview (n=50) retrospectively reported a mean CT score of 50.56 (s.d. 19.81), which was significantly higher than the mean CT score of 42.70 (s.d. 15.09) reported by cases who were not severely depressed at interview (n=122) (P = 0.026). However, interactions between PRS and CT were still significant after cases severely depressed at interview were excluded from the analysis (Table S9), suggesting that the interaction was not caused by a recall bias for negative events. This finding is consistent with two previous reports in the RADIANT UK sample, which indicated no evidence for recall bias due to low mood at interview .

**References**

**American Psychiatric Association** (1994). *Diagnostic and Statistical Manual of Mental Disorders 4th edition (DSM-IV)*. American Psychiatric Association: Washington DC.

**Battle, A., Mostafavi, S., Zhu, X., Potash, J. B., Weissman, M. M., McCormick, C., Haudenschild, C. D., Beckman, K. B., Shi, J., Mei, R., Urban, A. E., Montgomery, S. B., Levinson, D. F. & Koller, D.** (2014). Characterizing the genetic basis of transcriptome diversity through RNA-sequencing of 922 individuals. *Genome Research* **24**, 14-24.

**Beck, A. T., Steer, R. A. & Brown, G. K.** (1996). *Beck Depression Inventory – Second Edition Manual*. The Psychological Corporation: San Antonio, TX.

**Brugha, T., Bebbington, B., Tennant, C. & Hurry, J.** (1985). The List of Threatening Experiences: a subset of 12 life event categories with considerable long-term contextual threat. *Psychological Medicine* **15**, 189-194.

**Cohen-Woods, S., Gaysina, D., Craddock, N., Farmer, A., Gray, J., Gunasinghe, C., Hoda, F., Jones, L., Knight, J., Korszun, A., Owen, M. J., Sterne, A., Craig, I. W. & McGuffin, P.** (2009). Depression Case Control (DeCC) Study fails to support involvement of the muscarinic acetylcholine receptor M2 (CHRM2) gene in recurrent major depressive disorder. *Human Molecular Genetics* **18**, 1504-9.

**Farmer, A., Breen, G., Brewster, S., Craddock, N., Gill, M., Korszun, A., Maier, W., Middleton, L., Mors, O., Owen, M., Perry, J., Preisig, M., Rietschel, M., Reich, T., Jones, L., Jones, I. & McGuffin, P.** (2004). The Depression Network (DeNT) Study: methodology and sociodemographic characteristics of the first 470 affected sibling pairs from a large multi-site linkage genetic study. *BMC Psychiatry* **4**, 42.

**First, M. B., Spitzer, R. L., Gibbon Miriam & Williams, J. B. W.** (2002). *Structured Clinical Interview for DSM-IV Axis I Disorders* Biometrics Research, New York State Psychiatric Institute: New York.

**Fisher, H. L., Cohen-Woods, S., Hosang, G. M., Korszun, A., Owen, M., Craddock, N., Craig, I. W., Farmer, A. E., McGuffin, P. & Uher, R.** (2013). Interaction between specific forms of childhood maltreatment and the serotonin transporter gene (5-HTT) in recurrent depressive disorder. *Journal of Affective Disorders* **145**, 136-41.

**Fisher, H. L., Cohen-Woods, S., Hosang, G. M., Uher, R., Powell-Smith, G., Keers, R., Tropeano, M., Korszun, A., Jones, L., Jones, I., Owen, M., Craddock, N., Craig, I. W., Farmer, A. E. & McGuffin, P.** (2012). Stressful life events and the serotonin transporter gene (5-HTT) in recurrent clinical depression. *Journal of Affective Disorders* **136**, 189-93.

**Gaysina, D., Cohen-Woods, S., Chow, P. C., Martucci, L., Schosser, A., Ball, H. A., Tozzi, F., Perry, J., Muglia, P., Craig, I. W., McGuffin, P. & Farmer, A.** (2009). Association of the dystrobrevin binding protein 1 gene (DTNBP1) in a bipolar case-control study (BACCS). *American Journal of Medical Genetics. Part B, Neuropsychiatric Genetics* **150B**, 836-44.

**Keller, M. C.** (2014). Gene x environment interaction studies have not properly controlled for potential confounders: the problem and the (simple) solution. *Biological Psychiatry* **75**, 18-24.

**Levinson, D. F., Zubenko, G. S., Crowe, R. R., DePaulo, R. J., Scheftner, W. S., Weissman, M. M., Holmans, P., Zubenko, W. N., Boutelle, S., Murphy-Eberenz, K., MacKinnon, D., McInnis, M. G., Marta, D. H., Adams, P., Sassoon, S., Knowles, J. A., Thomas, J. & Chellis, J.** (2003). Genetics of recurrent early-onset depression (GenRED): design and preliminary clinical characteristics of a repository sample for genetic linkage studies. *American Journal of Medical Genetics. Part B, Neuropsychiatric Genetics* **119b**, 118-30.

**Lewis, C. M., Ng, M. Y., Butler, A. W., Cohen-Woods, S., Uher, R., Pirlo, K., Weale, M. E., Schosser, A., Paredes, U. M., Rivera, M., Craddock, N., Owen, M. J., Jones, L., Jones, I., Korszun, A., Aitchison, K. J., Shi, J., Quinn, J. P., Mackenzie, A., Vollenweider, P., Waeber, G., Heath, S., Lathrop, M., Muglia, P., Barnes, M. R., Whittaker, J. C., Tozzi, F., Holsboer, F., Preisig, M., Farmer, A. E., Breen, G., Craig, I. W. & McGuffin, P.** (2010). Genome-wide association study of major recurrent depression in the U.K. population. *American Journal of Psychiatry* **167**, 949-57.

**Nurnberger, J. I., Jr., Blehar, M. C., Kaufmann, C. A., York-Cooler, C., Simpson, S. G., Harkavy-Friedman, J., Severe, J. B., Malaspina, D. & Reich, T.** (1994). Diagnostic interview for genetic studies. Rationale, unique features, and training. NIMH Genetics Initiative. *Archives of General Psychiatry* **51**, 849-59; discussion 863-4.

**Shi, J., Potash, J. B., Knowles, J. A., Weissman, M. M., Coryell, W., Scheftner, W. A., Lawson, W. B., DePaulo, J. R., Jr., Gejman, P. V., Sanders, A. R., Johnson, J. K., Adams, P., Chaudhury, S., Jancic, D., Evgrafov, O., Zvinyatskovskiy, A., Ertman, N., Gladis, M., Neimanas, K., Goodell, M., Hale, N., Ney, N., Verma, R., Mirel, D., Holmans, P. & Levinson, D. F.** (2011). Genome-wide association study of recurrent early-onset major depressive disorder. *Molecular Psychiatry* **16**, 193-201.

**Uher, R., Perroud, N., Ng, M. Y., Hauser, J., Henigsberg, N., Maier, W., Mors, O., Placentino, A., Rietschel, M., Souery, D., Zagar, T., Czerski, P. M., Jerman, B., Larsen, E. R., Schulze, T. G., Zobel, A., Cohen-Woods, S., Pirlo, K., Butler, A. W., Muglia, P., Barnes, M. R., Lathrop, M., Farmer, A., Breen, G., Aitchison, K. J., Craig, I., Lewis, C. M. & McGuffin, P.** (2010). Genome-wide pharmacogenetics of antidepressant response in the GENDEP project. *American Journal of Psychiatry* **167**, 555-64.

**Wing, J. K., Babor, T., Brugha, T., Burke, J., Cooper, J. E., Giel, R., Jablenski, A., Regier, D. & Sartorius, N.** (1990). SCAN. Schedules for Clinical Assessment in Neuropsychiatry. *Archives of General Psychiatry* **47**, 589-93.

**World Health Organisation** (1998). *Diagnosis and Clinical Measurement in Psychiatry. A reference manual for SCAN*. World Health Organization: Geneva.

**World Health Organization** (1997). *Composite International Diagnostic Interview (CIDI), Version 2.1.* . World Health Organization: Geneva, Switzerland.
